# Supplementary material for: Chromatin-contact atlas reveals disorder-mediated protein interactions and moonlighting chromatin-associated RBPs
Source: Nucleic Acids Res. 2021 Dec 6;49(22):13092–107. doi: 10.1093/nar/gkab1180 (PMC8682780; doi:10.1093/nar/gkab1180)
Supplement: gkab1180_Supplemental_Files [file gkab1180_supplemental_files.zip › Supplementary Table legends.docx]

### **Supplementary Table S1: SPACE and SPACE-SICAP, related to Figure 2 and Figure 3**

- SPACE: Proteins quantified using SPACE by at least 1 forward and 1 reverse replicates
- SPACE_all_identifications: All proteins identified using SPACE experiments
- SPACE-SICAP: Proteins quantified using SPACE-SICAP by at least 2 replicates
- SPACE_SICAP_all_Identifications: All proteins identified using SPACE-SICAP experiments
- Total proteome: Proteins identified using total proteome of mouse ES cells (published data from Rafiee et. al. Mol Cell 2016 was re-analysed)
- DmChP: Proteins quantified by at least 2 EdU-plus replicates using DmChP (published data from Aranda S. et al, Science Advances 2019 were re-analysed)
- Chromatin pelleting: Proteins quantified by at least 2 replicates using chromatin pelleting (published data from van Mierlo G. et al Cell Stem Cell 2019 were re-analysed)
- Overlap of SPACE, RICK & CARIC: Proteins enriched using SPACE, RICK (Bao et al Nature Methods 2018) and CARIC (Huang et al PNAS 2018) as interactome of newly transcribed RNA.

### **Supplementary Table S2: SPACEmap_peptides, related to Figure 4**

- SPACEmap Peptides, Figure 4A: Peptides identified by SPACEmap, related to Figure 4A
- Overlap with SPACE, Figure 4B: Overlap of the crosslinked fraction (SPACEmap) and SPACE, related to Figure 4B venn diagram
- Mapped peptides, Figure 4B: Crosslinked fraction peptides mapped to a domain or region (Unique peptides), related to Figure 4B the upper bar
- Mapping details, Figure 4B-E: InterProScan results for SPACEmap crosslinked fraction, related to Figure 4B lower bar, Figure 4C, Figure 4D, Figure 4E
- SPACEmap verified caRBPs: 594 RBPs verified by SPACEmap and their chromatin-contact regions
- Clustered domains: Details of clustered domains, related to Figure 4D-E

### **Supplementary Table S3: Comparative SPACE and full proteome_2i_Serum, related to Figure 5**

- SPACE_2i_Serum: Comparative SPACE between 2iL and Serum conditions of mES cells. related to Figure 5
- Full_proteome: Full proteome analysis between 2iL and Serum conditions of mES cells. Related to Figure 5B (published data from Rafiee et a. Mol Cell 2016 was re-analysed)

### **Supplementary Table S4: Dazl_ChIP_iCLIP_SPACE, related to Figure 6**

- Dazl_ChIP-seq: Annotation of Dazl ChIP-seq peaks, related to Figure 6A
- Dazl_iCLIP-seq: Annotation of Dazl iCLIP peaks, related to Figure 6B
- Dazl_ChIP-SPACE: Dazl ChIP-SPACE, related to Figure 6D, E
